# Costs of receipt and donation of ejaculates in a simultaneous hermaphrodite

**DOI:** 10.1186/1471-2148-10-393

**Published:** 2010-12-25

**Authors:** Jeroen NA Hoffer, Jacintha Ellers, Joris M Koene

**Affiliations:** 1 Department of Animal Ecology, Faculty of Earth and Life Sciences, VU University, De Boelelaan 1085, 1081HV Amsterdam, The Netherlands

## Abstract

**Background:**

Sexual conflicts between mating partners can strongly impact the evolutionary trajectories of species. This impact is determined by the balance between the costs and benefits of mating. However, due to sex-specific costs it is unclear how costs compare between males and females. Simultaneous hermaphrodites offer a unique opportunity to determine such costs, since both genders are expressed concurrently. By limiting copulation of focal individuals in pairs of pond snails (*Lymnaea stagnalis*) to either the male role or the female role, we were able to compare the fecundity of single sex individuals with paired hermaphrodites and non-copulants. Additionally, we examined the investment in sperm and seminal fluid of donors towards feminized snails and hermaphrodites.

**Results:**

Compared to non-mating focal snails, reciprocating individuals as well as male and female copulants experienced a significant fecundity reduction (~40%) after, on average, 3.07 ± 0.12 copulations in their allowed roles (for donors 2.98 ± 0.16 copulations and for recipients 3.14 ± 0.12 copulations). In a single copulation, significantly more sperm was donated to partners that were restricted to mating in the female role than to hermaphrodites, while seminal fluid transfer was unaffected by recipient type.

**Conclusions:**

Our data indicate that the costs of mating in both sex functions are high in *L. stagnalis*. This conclusion is based on fecundity data collected separately for male and female copulants. Male mating costs result from investment in expensive ejaculates, composed of sperm and seminal fluid. For female copulants, fecundity reduction correlated with transferred sperm numbers in the first copulation, while differences in transferred quantities of seminal fluid were not detected. These findings may point toward a "sperm effect" as a novel feature of pond snail reproductive ecology. In conclusion, sex allocation and sexual conflict both contribute to decreased female fecundity in pond snails.

## Background

In contrast to earlier views of reproduction as a cooperative affair between two mating partners, it has become evident that sexual encounters between males and females are commonly accompanied by conflict between sexually interacting partners [1]. A basic understanding of this phenomenon can be found in Bateman's principle, which holds that a female's reproductive output is primarily limited by access to resources while male fitness is primarily limited by access to female gametes [2,3]. The most important implication of this is that females are selected to be choosy about whom to mate with, while males can profit from mating with many females. Therefore, with the exception of strict monogamy, the genetic interests and optimal reproductive strategies of mating partners will rarely coincide.

Under these circumstances a trait that is advantageous for one mating partner but harmful to the other can fuel repeated cycles of adaptation and counter-adaptation in the two sexes, i.e. a coevolutionary arms race [4]. The last decade has seen many contributions showing that such sexual antagonism between the sexes can severely impact the evolution of reproductive behavior, physiology and morphology in animals [5-7]. For instance, conflict over mating in water striders has led to the evolution of harassment behaviors and grasping structures in males and anti-grasping traits in females [8]. Although sexual conflict in plants is less extensively studied, here supporting evidence is also accumulating [9,10]. In addition to antagonistic traits in males and females, competition between males can indirectly cause sexual conflict when traits beneficial in sperm or pollen competition - as a corollary - harm females and lower their fitness [11-14]. For example, early induction of stigma receptivity by pollen donors promotes avoidance of pollen competition in *Collinsia heterophylla*, but is associated with lower seed set of pollen recipients [15]. However, investment of limiting resources into expensive pollen or ejaculates [16] that maintain sperm competitiveness has also been demonstrated to come at non-trivial costs to other life-history traits in males [17-19]. It is therefore not surprising that males modulate ejaculate size according to sperm competition risk, which can even lead to sperm-limited females [20]. Hence, the expression of traits beneficial in sperm competition and female manipulation, e.g. male accessory gland proteins, is costly to their bearers, such that males can become limited in the number of fertilizations they can secure.

Ultimately, the evolutionary outcome of sexual conflict for both sexes depends on the relation between costs and benefits of mating interactions, which define the fitness pay-off [4]. Benefits are typically expressed in additional eggs, sired offspring or enhanced offspring quality, which can all increase an individual's fitness. Costs can also take several forms. First, costs of production that result from, for instance, sperm and seminal fluid synthesis. Second, costs of manipulation result when receipt of manipulative compounds shift reproductive investment away from the optimum. Third, costs of collateral harm result from loss of resources, e.g. when received compounds (or their metabolites) need to be detoxified. All costs accumulate over an individuals' lifetime and lead to loss of fitness.

In gonochorists (i.e. separate sex species), costs and benefits are normally estimated separately for males and females, and can be inferred by comparing populations or closely related species under different selection pressures [21,22]. Importantly, direct comparison of costs of mating for both sexes requires a common measure of reproductive investment, which is problematic in separate sex species. In hermaphrodites, however, a basic assumption of sex allocation theory is the existence of a fixed budget for reproduction, thus resources spent on the male function will trade off against female reproductive investment and vice versa. As a consequence, costs of donation and subsequent *de novo *synthesis of ejaculates can be detected via a decrease in female reproductive investment. Second, the cost of manipulation of reproductive investment, i.e. sex allocation, can be traced by proxies of fitness in both reproductive functions. Again, when predictions of sex allocation theory hold, manipulation by donors could for example, result in an increase in egg production, and a concurrent decrease in male reproductive investment. Third, as collateral harm usually affects both sexes, the costs of harm can be traced through a decrease in fecundity, but since the male function should be affected too, ideally, ejaculate investment should be measured as well.

The flexibility of sex allocation in hermaphrodites is of great importance since it allows them to adapt quickly to local changes in reproductive opportunities. However, plasticity in sex allocation increases vulnerability to exploitation by mating partners. The latter occurs because selection favors sperm donors (hereafter donors) that transfer ejaculates that increase egg production in receiving partners (hereafter recipients), creating potential for sexual conflict over sex allocation [23,24], which is predicted to evolve readily [25]. Costs of such manipulation fall in the second category mentioned above, and may be detected through decreased investment in the male function or increased egg output and/or enhanced growth (size advantage model [26]). Despite the attention for sexual conflict in hermaphrodite mating systems, data on the costs of expression of antagonistic traits is rare (or involved the concurrent elimination of both sexual functions [27]), although several authors point out that such data would be essential for determining the evolutionary consequences of antagonistic traits [4,28,29].

The hermaphroditic pond snail *Lymmaea stagnalis *has been used as a model to study reproductive ecology [e.g. [30-32]]. De Visser et al. [31] found that snails that were allowed to copulate produced half as many eggs as non-copulating individuals, despite the presence of stored allosperm in all snails. This led them to conclude that in non-copulants male resources were re-allocated to the female function, as sex allocation theory predicts [23,33]. This study has been considered the most clear-cut demonstration of resource re-allocation between male and female function, and thus a direct trade-off as predicted by sex allocation theory. Quite to the contrary, a study by Van Duivenboden et al. [30] showed that inhibition of egg laying occurs upon copulating as a female, suggesting that compounds in the ejaculate may be responsible. This idea is corroborated by recent work [34,35] showing that seminal proteins transferred during copulation induce a delay in egg mass production.

To distinguish between the two hypotheses for the decreased fecundity upon copulation, we performed two experiments using *L. stagnalis *to determine the costs associated with copulation and the mechanism underlying loss of fecundity. In the first experiment we restricted the roles in which snails could copulate. The costs of mating as a male and a female were expressed in a common measure of investment, namely the decrease in egg production compared to non-mating animals. In the second experiment, we specifically assessed the investment in ejaculates (i.e. male investment) by determining the number of sperm and amount of seminal fluid transferred during a single copulation. Taken together, these experiments provide the first quantification of the costs of ejaculate receipt and donation in a simultaneously hermaphroditic animal.

## Results

### Effect of mating role on egg production

**Figure 1:**
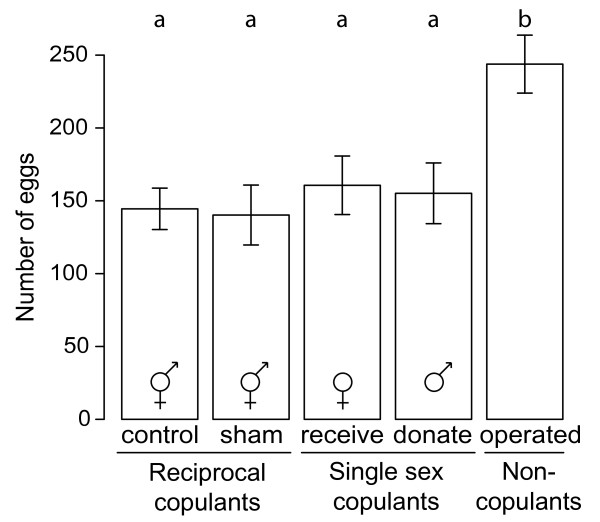
**Fecundity of *Lymnaea stagnalis *individuals in restricted mating roles during five days**. Fecundity during five days for *Lymnaea stagnalis *individuals restricted to experimentally allowed mating roles. "Control" and "Sham" treatments include reciprocally mating focals ("Reciprocal copulants"), whereas the "Receive" and "Donate" treatment' focals are restricted to mating as a recipient and as a donor, respectively ("Single sex copulants"). In the "Operated" treatment focals do not mate at all ("Non-copulants"). The symbols depicted in the bars denote the imposed mating role(s) of the focal individuals in the different treatments. The bars indicate mean ± 1 SE. Tukey post-hoc results are shown above the bars.

**Figure 2:**
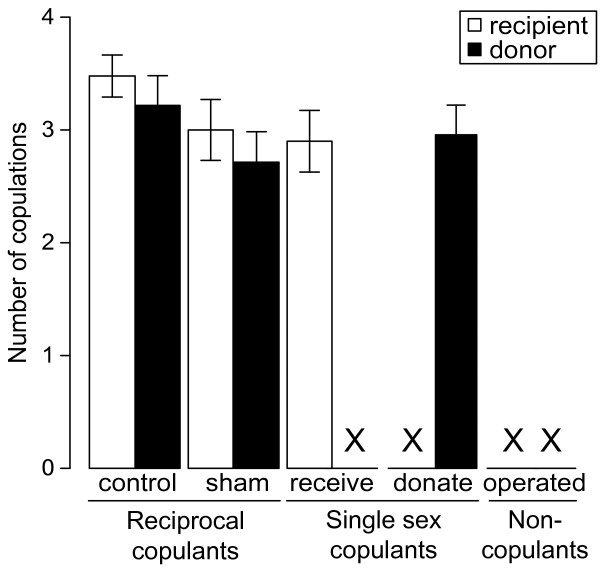
**Number of copulations in experimentally imposed mating roles**. The number of copulations of *Lymnaea stagnalis *in the imposed mating role(s). The bars indicate the mean number of copulations as a recipient (white bars) and as a donor (black bars) ± 1 SE. Note that an X indicates prohibited mating roles (missing values), that were excluded from statistical analysis.

During five days pairs of pond snails were observed for mating activity and fecundity. Between treatments there was no difference in shell length, which is tightly correlated with body size (ANOVA: *F*_4,106 _= 0.64, *P *= 0.64). The overall difference in fecundity was significant for treatment but not for size (ANCOVA on square root transformed fecundity data: *F*_4,106 _= 7.94, *P *< 0.001 and *F*_1,106 _= 0.12, *P *= 0.73, treatment and shell length, respectively). The non-significant interaction term was dropped from the model. The treatment effect was entirely due to the higher fecundity of animals in the "Operated" treatment compared to the other 4 treatments, whereas none of the other groups differed significantly (Figure 1). The dry weight per egg did not differ significantly between treatments (ANOVA: *F*_4,106 _= 1.90, *P *= 0.12). In addition, there was no significant difference between the number of eggs per egg mass (ANOVA: *F*_4,106 _= 1.76, *P *= 0.14), but the number of egg masses laid was different between treatments (ANOVA: *F*_4,106 _= 17.79, *P *< 0.001). This difference was due to more egg masses being produced in the "Operated" treatment (Tukey post-hoc test: *P *< 0.05). Interestingly, the number of copulations in the allowed roles did not differ between treatments (ANOVA on number of copulations: *F*_2,64 _= 0.80, *P *= 0.45 and *F*_2,61 _= 2.22, *P *= 0.12; for donors and recipients, respectively). Thus, animals that were restricted to one mating role did not perform that role more often than reciprocating hermaphrodites (Figure 2). On average, individuals mated 2.98 ± 0.16 times as a donor and 3.14 ± 0.12 times as a recipient, when allowed. Since animals used in the experiment were all of the same age cohort and consumed equal amounts of lettuce, we assumed that the costs of bodily maintenance and growth were also equal, based on the tight relationship between shell length and body weight [36].

### Ejaculate donation to hermaphrodites and females

**Figure 3:**
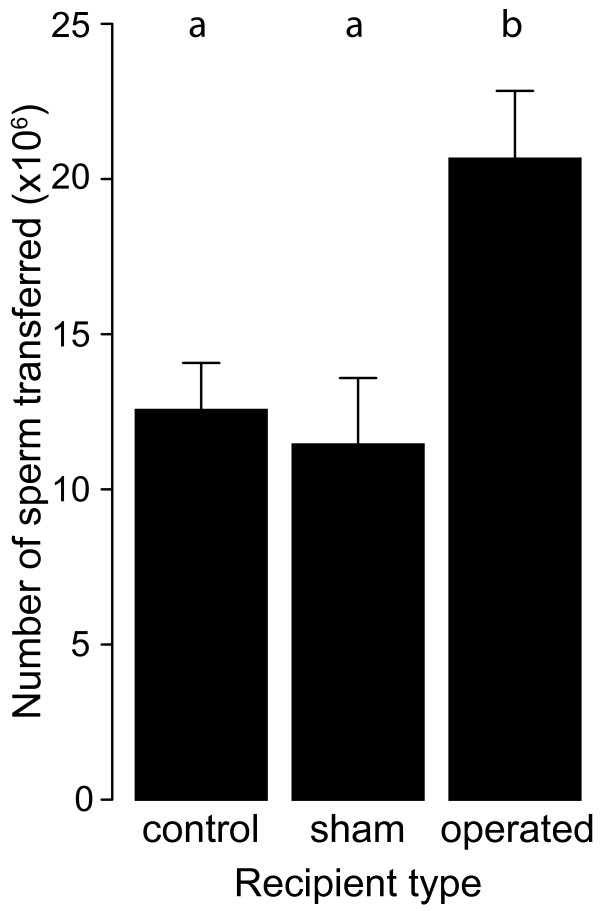
**Sperm transfer to hermaphrodite, sham and operated individuals**. The number of sperm transferred during copulation of *Lymnaea stagnalis *towards three categories of recipients. Sperm was recovered from the female tract of the recipients directly after mating. The bars represent mean ± 1 SE with post-hoc results indicated above the bars.

Directly after insemination by standardized donors we determined the number of sperm transferred to either unoperated, sham operated or operated recipients. The differences in the amount of sperm transferred between treatments was highly significant (ANOVA on square root transformed sperm counts: *F*_2,38 _= 9.39, *P *= 0.001, Figure 3). This was due to higher numbers of sperm transferred to surgically feminized recipients (Post-hoc: *P *< 0.001, Figure 3), whereas the other treatments did not differ from each other. The sizes of both recipients and donors were not significantly correlated to numbers of sperm transferred (linear regression: *r^2 ^*< 0.01, *F*_1,39 _= 1.03, *P *= 0.32 and *r^2 ^*= 0.02, *F*_1,39 _= 0.04, *P *= 0.84, recipients and donors, respectively). Shell length was also not significantly different among treatments (ANOVA: *F*_2,38 _= 0.05, *P *= 0.95).

**Figure 4:**
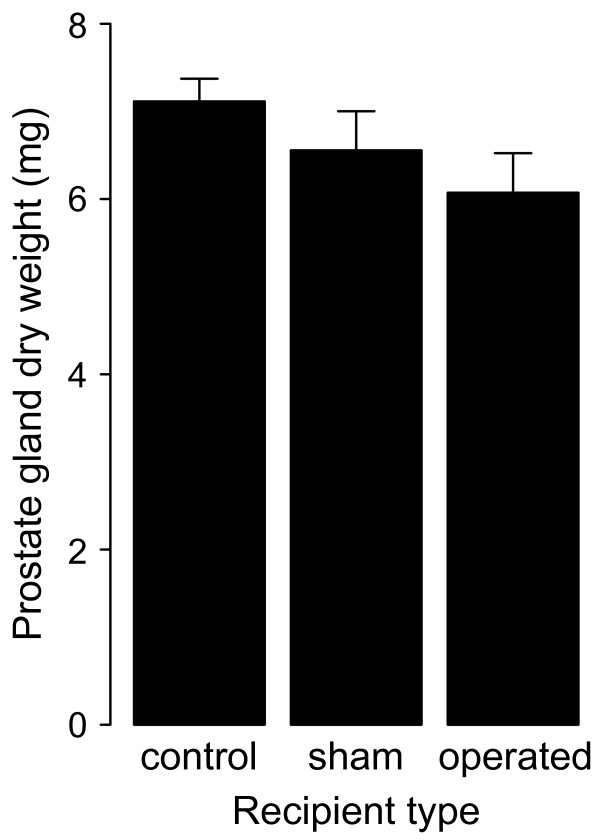
**Dry weight of donor prostate glands**. Prostate gland dry weights of *Lymnaea stagnalis *excised from donors directly after copulation with three categories of recipients. A lower prostate mass indicates the donation of more seminal fluid during copulation. The bars represent mean ± 1 SE.

In addition to sperm, prostate gland products added to production costs of ejaculates. Prostate glands dry weights were proportional to body size (linear regression: *r^2 ^*= 0.24, *F*_1,72 _= 23.21, *P *< 0.001). After removing the non-significant interaction term (treatment × mating role) from the model, we found that mating role (donor or recipient) had a significant effect on prostate dry weight, while treatment did not (ANCOVA on residual prostate dry weight: *F*_1,70 _= 4.50, *P *= 0.04 and *F*_2,70 _= 0.33, *P *= 0.72, for mating role and treatment, respectively). Thus, donors from different treatments did not have different prostate weights after a single insemination (Figure 4). Overall, donor prostates weighed approximately 14 percent less than recipient prostates (6.59 ± 0.29 mg vs. 7.62 ± 0.26 mg). Thus, a considerable amount of stored accessory gland secrete was transferred during a single copulation. Based on these findings we concluded that although sperm numbers were modulated depending on the type of recipient, the amount of seminal fluid transferred (as measured via prostate weight) during copulation was not.

## Discussion

We found that single sex-acting individuals experienced a similar decrease in fecundity as did reciprocally mating individuals. From the perspective of the female function this suggests receipt of ejaculates to cause reduced fecundity, because reciprocals received an equal number of ejaculates compared to females. In addition, reciprocals mated on average the same number of times as donors did. Without knowledge about the cost of ejaculate donation this may suggest that all of the observed reduction in egg production after mating is due to receipt of an ejaculate, as has been proposed before [30]. Surprisingly, however, for animals restricted to the male role, egg production was decreased to the same extent as in females and reciprocals. Since focal males did not receive any ejaculates, any decrease in fecundity can only be attributed to copulations as donors and suggests a re-allocation between male and female function. However, focal males donated the same number of times as reciprocals did, which seems incompatible with the observed costs of mating in reciprocals.

This paradox highlights the importance of also quantifying ejaculate investment to different recipient types. In fact, we found that sperm donation in the first copulation after an isolation period was twice as high towards imposed females compared to hermaphrodites. Provided that these sperm investment patterns persist over consecutive matings, the increased investment in these ejaculates provides a possible mechanism for the observed decrease in fecundity of male acting snails compared to reciprocals. For reciprocally mating snails, on the other hand, ejaculate receipt as well as ejaculate production and delivery costs may be responsible for decreased fecundity in mating individuals. Based on these results we conclude that Van Duivenboden et al. [30] and De Visser et al. [31] were both partly correct.

The results of our second experiment suggest that the main costs of ejaculates arise from enhanced investment in sperm and not from seminal fluid investment. Although this outcome is only based on the costs of a single copulation, it is surprising for three reasons. First, previous work on *L. stagnalis *showed that inhibition of egg laying is the result of at least one seminal protein and not of the presence of sperm [37]. Therefore, one may expect that the amount of seminal fluid would be modulated instead of sperm. However, the dry weight of donor prostates is independent of recipient type. In this respect it is a tentative idea that not the total volume, but rather the relative quantities of transferred proteins within an ejaculate are modulated. Such strategic allocation of seminal peptides has been shown to occur in *Drosophila melanogaster *[38] indicating that such mechanisms could also operate in other species where post-copulatory sexual selection is important.

Second, although it is known that pond snails are capable of strategic sperm donation [39], the result that more sperm are transferred to imposed females compared to hermaphrodites is unexpected. This is simply because feminized snails are not more fecund than hermaphrodites when kept in isolation [30,35]. Donors are therefore unlikely to benefit from increased sperm investment towards operated snails. Although speculative, it is in principle possible that surgery interferes with a cue used by donors for partner assessment. This may alter the donor's expectation of egg load and/or risk of sperm competition, but an active role of the recipient in accepting larger ejaculates cannot be ruled out. Nonetheless, sperm allocation was found to be tailored according to recipient type. Subsequent copulations with other recipients of the same type may yield similar sperm investments [40]. Conversely, repeated large donations could also quickly deplete sperm stores, resulting in decreasing investments [20]. It therefore remains to be tested in pond snails whether sperm donation in subsequent copulations continues to follow this pattern of male investment.

Third, in combining the results from the two experiments it appears that imposed females (in the "Receive" treatment) suffer decreased fecundity from receipt of increased numbers of sperm. This is the first indication that a "sperm effect" may be present in *L. stagnalis*. The previous absence of a sperm effect [37] is possibly a consequence of the methods used. Namely, in the latter paper, sperm was recovered from excised sperm storage organs and mixed with single purified prostate proteins before experimental insemination. This practice excludes any male compound that may originate from either the sperm duct or the vas deferens and penial complex, which may be present in naturally transferred ejaculates. Especially the sperm duct seems relevant in this respect, since its epithelium contains three types of excretory cells that contain proteinaceous compounds [41]. Similarly, although in fruit flies the accessory male gland products have received most attention, their ejaculatory duct and ejaculatory bulb also add molecules to seminal fluid [42,43]. Clearly, these additional secretions deserve closer investigation with respect to their functions in animal reproductive biology.

In female copulants, net egg investment is decreased, since fecundity losses are not balanced by increased investment per offspring (i.e. dry weight per egg). This implies that the fitness of both mating partners is affected, which is indicative of a harmful strategy. Inflicted harm can be adaptive if female responses to harm benefit the donor. Alternatively, harm is collateral when traits involved in intraspecific competition also have deleterious effects on females as a side-effect [4,44]. Under the experimental conditions tested, we find no indication that pond snails increase the remating interval or egg output which would suggest the harm to be adaptive [but see [45]]. However, the fact that pond snails are highly promiscuous [32] and sperm of multiple partners can be stored for three month after insemination [46], harm could arise collaterally from adaptations to sperm competition.

## Conclusions

In the hermaphrodite *L. stagnalis *we find high costs of mating for both mating roles. From the decrease in fecundity of imposed males we infer that donor costs result from investment in sperm and seminal fluid. Female copulants are negatively affected by the receipt of ejaculates. Interestingly, the fecundity reduction correlated with sperm numbers in the first copulation, while differences in seminal fluid quantities were not found. This "sperm effect" may be a novel feature of pond snail reproductive ecology. In addition, we conclude that two earlier hypotheses on the causes of fecundity decrease upon mating are both correct [30,31], depending on the mating role an individual snail assumes.

Although the current study lacks measures of lifetime fecundity, we show that the costs of mating in both sex roles are considerable in our model system. Such costs are important determinants of reproductive dynamics in populations, since they affect fitness pay-offs. Future studies could combine mating costs with proxies for fitness, so that fitness pay-offs in units of investment via both reproductive functions in hermaphrodites can be used to evaluate the impact of sexually antagonistsic interactions and harm. The quantification of the costs of receipt and donation of ejaculates, as we present here, is therefore a necessary step, by providing insight into the economics of sexual conflict in a hermaphrodite.

## Methods

### Study species

*Lymnaea stagnalis *is a common pond snail species in the northern hemisphere, where it resides in freshwater lakes, ponds and ditches. Pond snails are hermaphroditic but mate unilaterally, i.e. one mating partner performs the male sexual role during copulation and the other the female role. During copulation copious amounts of sperm and seminal fluid from the prostate gland are transferred to the recipient snail. In this promiscuous species, received sperm from several donors can be stored for up to 3 months [46]. Although individuals also carry autosperm, which they can use for self-fertilization, allosperm is preferentially used for fertilization, resulting in complete out-crossing of offspring after receipt [47].

The motivation to act as a donor is highly correlated with the volume of the prostate gland. A part of the penial nerve (branch NP1) that runs from the prostate to the central nervous system informs the brain about the filling status of the prostate [48]. Mating depletes seminal fluid reserves in the prostate gland, which are replenished after 8 days of abstinence [49]. When both mating partners are motivated to donate, mating roles can be swapped after the first insemination [50].

### Effect of mating role on egg production

Adult animals from a three-months-old cohort were collected from our mass rearing culture and housed individually in perforated plastic jars in a large tank with running low-copper water under standard lighting conditions (light:dark = 12:12). Every day after isolation each snail received one disc of lettuce of 19.6 cm^2^. This amount was slightly below their maximum intake rate, so that all lettuce was consumed each day [36]. After acclimatizing for four days to experimental conditions the snails were checked for egg laying capability and were randomly assigned to one of three experimental procedures. We anesthetized one group by injecting ~2 ml of 50 mM MgCl_2 _using a syringe. Subsequently, we cut away (lesioned) part of the vas deferens which, crucially, runs parallel with part of the penial nerve (NP1) through the skin. This surgery removes the motivation to mate in the male role [48] and as a result turns operated individuals into females (although male organs remain intact). A second group was anesthetized and cut in the skin directly above the vas deferens, i.e. sham operated. These animals remained fully functional in both reproductive roles. A third group was left intact. In addition to these procedures, a numbered tag (Het Bijenhuis, The Netherlands) was glued to the shell of half of the animals of all groups for identification. After the glue dried all snails were returned to isolation under the conditions mentioned above.

On the eighth day after isolation (when male motivation reaches its maximum) animals from the three different experimental procedures (222 animals in total) were assigned to five treatment groups. Two treatments were made by pairing two functional hermaphrodites, with the difference that one group consisted of two unoperated individuals ("Control") and the other of two sham operated individuals ("Sham"). Both pair types had one focal snail and will be jointly referred to as reciprocal copulants (reciprocals for short) since they were given the opportunity to mate in both sexual roles. For the third treatment an operated focal was paired to an unoperated individual. This restricts both individuals to copulating in one role only, since the focal is effectively a female and cannot reciprocate as a donor. Therefore, this was the "Receive" treatment, where we referred to focals simply as females. The fourth treatment was identical to the third, except that here the focal was the unoperated hermaphrodite. Since focal individuals were restricted to donating and never received from their partner (a female), this was the "Donate" treatment. Focals were simply called males. Finally, the "Operated" treatment consisted of two operated individuals one of which served as focal. Since both snails lacked motivation to mate as a male, this treatment is characterized by a general lack of copulatory activity, hence focal individuals are referred to as non-copulants. A schematic overview of the make-up of these five experimental treatments is given in Table 1.

**Table 1:** Schematic overview of pairing types of Lymnaea stagnalis individuals in five experimental treatments.

Treatment	Pairing type		Description
	Focal	Partner	
Control	H	H	Reciprocal copulation, focals receive and donate.
Sham	H	H	Reciprocal copulation, focals receive and donate.
Receive	F	H	Unilateral copulation, focals restricted to receiving.
Donate	H	F	Unilateral copulation, focals restricted to donating.
Operated	F	F	No copulation, focals neither receive nor donate.

Capital letters denote hermaphrodites (H) and surgically feminized individuals (F). Surgery involves lesioning a branch of the penial nerve (NP1) in snails and removes the motivation to mate as a male (see text for details). Therefore, combinations of H and F result in pairs that can only copulate in one mating role: H as donor and F as recipient. In FF pairs, male function is lacking and copulatory activity is absent. HH pairs denote normally mating functional hermaphrodites.

Each day, all pairs (including operated pairs) were observed until mating activity ceased (1.5 - 8 hours) before they were returned to isolation for the remainder of the day and received food. This protocol was the same for the five days of the observational period. High mating rates were ensured by making use of the Coolidge effect, i.e. the motivation to inseminate novel partners over previous partners [32]. Under these circumstances, sperm is transferred in nearly all copulations as has been made plausible previously [51]. Each day focals were paired with different mating partners, which were circulated within treatments. For the focal individuals mating roles were noted and egg masses produced during both day (immediately upon laying) and night (next morning) were collected for egg counting. Egg masses laid by non-focals were discarded as soon as they were produced. Counting of eggs was greatly facilitated by placing egg masses in a vial containing 70% ethanol, which renders the egg bodies opaque within an hour. After counting, egg masses of each focal were pooled in a pre-weighed collection tube. After freeze drying, the dry weight of each egg mass was determined to the nearest 0.02 mg on a closed microbalance (Sartorius, model 1712 MP8, Göttingen, Germany). By dividing the total dry weight by the total number of eggs laid by an individual we calculated the investment per egg. Directly after the experiment shell length of focal individuals was measured with calipers to the nearest 0.1 mm.

### Ejaculate donation to hermaphrodites and females

Adult snails taken from the mass rearing culture were isolated and maintained as described above. After checking for egg laying capability on the fourth day after isolation, half of the 94 snails were randomly distributed into three groups that were either surgically feminized, sham operated or left intact, following the same procedures as described in the previous section. The other half of the experimental snails remained in isolation without manipulation and would serve as standardized donors. Bee tags were glued on the shells for identification.

On the eighth day after initial isolation pairs of snails were assigned to one of three treatment groups. The "Control" treatment consisted of an unoperated snail paired to another unoperated individual that was the prospective donor. The "Sham" treatment consisted of a sham operated snail paired to an unoperated donor. In the "Operated" treatment unoperated donors were paired to operated individuals. All pairs were observed and the mating role of focal snails was noted. As soon as a focal had received sperm (none mated first as a male), both donor and recipient were anesthetized and shell lengths were determined. Subsequently, the shell of the recipient was removed and the skin was cut open using small surgical scissors, taking care not to puncture the underlying sperm-filled ducts. The female ducts (vaginal duct, truncus bursae/pedunculus and oviduct) were dissected out as a whole and placed in a 1.5 ml vial containing 200 μl of saline. After vortexing for 30 s the female tissue was transferred to another vial containing 200 μl of saline and vortexed again. This was repeated a third time after which the tissue was discarded and the three vials were pooled (600 μl in total). The sperm in each sample was counted in quadruple using a Neubauer counting chamber (0.1 mm depth by 0.0025 mm^2 ^surface area). A more detailed description of the protocol can be found elsewhere [39]. In addition, the prostate glands of both the recipient and the donor were dissected out and placed in separate pre-weighed vials after which the dry weights (after freeze drying) were determined.

### Statistical analyses

All statistical analyses were performed in R (version 2.8.1). When the data were not normally distributed they were transformed to suit model assumptions. In the experiment where we looked at fecundity upon mating, one focal in the "Donate" treatment mated as a female, and was therefore excluded from the analyses. In the sperm transfer experiment, data from six recipient snails were excluded from the analysis (~10%, two in each treatment). These individuals had appeared to be copulating but no sperm was recovered from the female ducts.

## Authors' contributions

JNAH and JMK have conceived and designed the study and carried it out, performed the statistical analyses and drafted the manuscript. JE was instrumental in conceiving the study, contributed to the statistical analyses and participated in drafting the manuscript and revised it critically for important intellectual content. JNAH coordinated all steps of the process. All authors have read and approved the final manuscript.
